# Engineering rhizobacterial communities for soil and plant health

**DOI:** 10.1099/mic.0.001748

**Published:** 2026-08-07

**Authors:** Elena Garcia-Perez, Louis Perrin, Jacob G. Malone, Catriona M. A. Thompson, Sarah Guiziou

**Affiliations:** 1 Earlham Institute, Norwich Research Park, Norwich NR4 7UZ, UK; 2 John Innes Centre, Norwich Research Park, Norwich, NR4 7UH, UK; 3 University of East Anglia, Norwich Research Park, Norwich, NR4 7TJ, UK

**Keywords:** bacterial communities, EngComs, engineering biology, plant–bacteria interaction, rhizobacteria, SynComs

## Abstract

Rhizobacteria play a central role in supporting plant growth, contributing to nutrient acquisition, stress tolerance and disease suppression. Harnessing and improving rhizosphere microbial communities therefore represents a promising avenue towards more sustainable agriculture. Recent advances in microbiome ecology and synthetic biology have enabled the rational design of microbial consortia. Synthetic communities are widely used as tractable models to study ecological interactions and are increasingly explored as biofertilizers and biocontrol agents. Here, we define engineered microbial communities (EngComs) as microbial consortia augmented with strains carrying synthetic genetic circuits. These systems extend SynCom approaches by enabling programmable functions, such as intercellular communication, division of labour, biosensing and controlled nutrient mobilization, ultimately improving functional stability in complex environments. Beyond bacteria–bacteria interactions, we highlight emerging strategies to engineer plant–microbe interfaces through synthetic signalling pathways and multi-input genetic circuits that enable context-dependent responses. Despite this progress, the engineering of rhizobacteria for real soil environments remains at an early stage. Most systems are still characterized in simplified or artificial conditions, and key challenges persist, including environmental complexity, genetic stability, biocontainment and regulatory constraints. Addressing these limitations will be essential to translate engineered functions from laboratory settings to the field. Overall, continued integration of synthetic biology with ecological and biophysical understanding of the rhizosphere will pave the way for programmable plant–microbe systems, offering new opportunities to enhance crop productivity while reducing environmental impact.

## Introduction

Agriculture is entering a period of unprecedented pressure. Climate change is reshaping temperature regimes, water availability and the ecological networks that sustain crop productivity, while a growing global population intensifies the demand for resilient and sustainable food systems [1, 2]. Central to resolving this challenge are the microbial communities that live within the soil, specifically the rhizosphere communities [3]. The rhizosphere is the dynamic soil interface where plants and microbes interact to regulate nutrient acquisition, stress tolerance and overall plant health [4]. Harnessing these microbial partnerships offers a powerful avenue to reduce chemical inputs and promote soil regeneration. Rhizobacteria are increasingly being explored as biofertilizers and biostimulants in this context [5, 6].

Microbial interactions play a crucial role in plant health and productivity, influencing soil physicochemical properties [7], regulating carbon and nitrogen cycles [8, 9] and supporting plant health. In particular, beneficial members of the rhizosphere and endophytic microbes can boost plant growth, biocontrol and stress resilience through diverse mechanisms [10, 11]. Microbial recruitment by the plant can convey direct benefits to both the plant and surrounding ecosystem, for example through the generation of suppressive soils that sustainably inhibit root pathogen activity [12, 13]. Plants actively recruit and shape the microbial community in the rhizosphere by secreting a diverse array of primary and secondary metabolites into the surrounding soil [14–18]. This microbiome selection takes place both via direct plant–microbe signalling and by modifying the availability of key nutrients [15, 19, 20]. The biocontrol and biostimulation characteristics of root-associated bacteria are highly diverse, underpinned by extensive, untapped biosynthetic potential [10, 15, 21] and subject to strong selective pressure, with both plant root location [22] and plant genetics [12, 13, 15, 23] acting as major drivers of microbial selection.

Rhizosphere microbial communities play a key role in plant health and productivity, making them attractive targets for intervention in sustainable agriculture. Engineering these systems offers the possibility to enhance nutrient acquisition, improve stress resilience and suppress disease in a programmable and scalable manner. Bacteria are particularly well suited for this purpose due to their short generation times, genetic tractability, broad host-range tools and increasingly predictable design frameworks [24]. Existing applications, such as nutrient-solubilizing strains, biosensors and biocontrol agents, demonstrate the potential of microbiome-based engineering approaches [25, 26]. However, these applications typically rely on native regulatory architectures and remain limited in their ability to perform robust, context-dependent functions across heterogeneous soil environments.

Advances in engineering biology now provide a foundation to move beyond these limitations by enabling the design of synthetic gene circuits in the bacteria with programmable sensing, information processing and intercellular communication. This article outlines how engineering approaches can expand the utility of rhizobacteria and pave the way for next-generation microbial interventions in agriculture. We first introduce strategies to construct and study synthetic communities (SynComs), and how these can be extended into engineered microbial communities (EngComs). We then discuss emerging approaches to programme plant–microbe interactions and highlight advances in engineering rhizobacteria to sense, record and respond to environmental cues. Finally, we consider the key challenges for field deployment, including environmental complexity, circuit robustness, biocontainment and regulatory constraints.

## Synthetic communities as models of rhizosphere diversity and biofertilizers

The high complexity of natural microbial ecosystems makes it difficult to unpick the inter- and intraspecies interactions that underpin the success of these communities. SynComs are defined here as experimentally assembled consortia composed of a limited number of well-characterized microbial strains. SynComs are powerful tools that allow, as example, these complex interactions to be understood and the development of multispecies bioinoculants [27, 28] for agriculture use. The relative simplicity and defined nature of SynComs, compared to natural microbial communities, allow for molecular and ecological interactions to be investigated in a highly reproducible and reliable manner [28]. Although well-designed SynComs can be an excellent model to interrogate the ecological dynamics of microbial ecosystems [27–29], they can never entirely recapitulate the complexity and multikingdom diversity of the natural microbiota [28]. Therefore, each SynCom should represent the aspects of the ecosystem that are important to either the area of study or its application to agriculture [28].

The broad principle of SynCom design is to assemble representative members to mimic the taxonomic and functional traits of a natural community [27, 30]. Whilst multiple strategies can be utilized to successfully design SynComs, these can be broadly categorized into ‘bottom-up’ and ‘top-down’ approaches. Both approaches have proven effective for investigating plant–microbe interactions and for developing applications such as crop protection, biocontrol and biofertilization.

Bottom-up approaches aim to recapitulate community interactions with minimal levels of complexity [28]. These approaches use individual species or strains as building blocks to recapitulate microbial interactions using genetically and phenotypically characterized strains [28, 30]. These SynComs often contain a small number of strains from a specific host or are assembled from well-characterized model strains that may never naturally co-occur in the environment [28, 30]. Bottom-up approaches have been used to successfully design SynComs to improve tolerance to abiotic stressors, with SynComs providing improved drought tolerance in *Arabidopsis thaliana* [31] and in barley [32]. Bottom-up SynComs have also been designed to protect plants against microbial pathogens, including providing protection against *Fusarium* sp. infection in multiple plant species [33–36], *Ralstonia solanacearum* [37–39] and *Rhizoctonia solani* [40] infections.

By contrast, top-down approaches attempt to more closely mirror the diversity and complexity of natural environments [30]. Rather than reconstructing communities from a small number of representative members, top-down approaches start from complex environmental samples and selectively enrich or reduce them based on defined criteria. This can involve applying selective pressures (e.g. nutrient regimes or stress conditions) or using sequencing-informed selection of community members. As a result, top-down strategies retain emergent interactions and functional redundancy that are often lost in simplified systems [28, 30]. Top-down strategies have been successfully used to identify keystone taxa and core functional traits [28, 30, 41], while maintaining community-level behaviours observed in nature [29]. A notable example is the generation of plant-associated SynComs from host-specific, well-characterized culture collections, such as for *A. thaliana:* At-RSPHERE [42] and At-LSPHERE [43] and for *Lotus japonicus:* Lj-SPHERE. These resources enable the construction of reproducible SynComs that approximate native rhizosphere or phyllosphere community composition, respectively, facilitating mechanistic studies of host–microbe interactions [44], host preference, microbial population dynamics [43], disease suppression [29, 45] and increasing crop growth [29] and yield [45].

Although SynComs can be used to great effect, their design is subject to important limitations. While bottom-up approaches risk missing higher-order interactions, top-down strategies frequently compromise predictability and reproducibility [30]. These two approaches are therefore best viewed as complementary, and their integration, supported by emerging computational and ecological modelling frameworks (reviewed here [46]), offers a promising path towards more robust and rational SynCom design (explored fully here [47]).

**Fig. 1.**
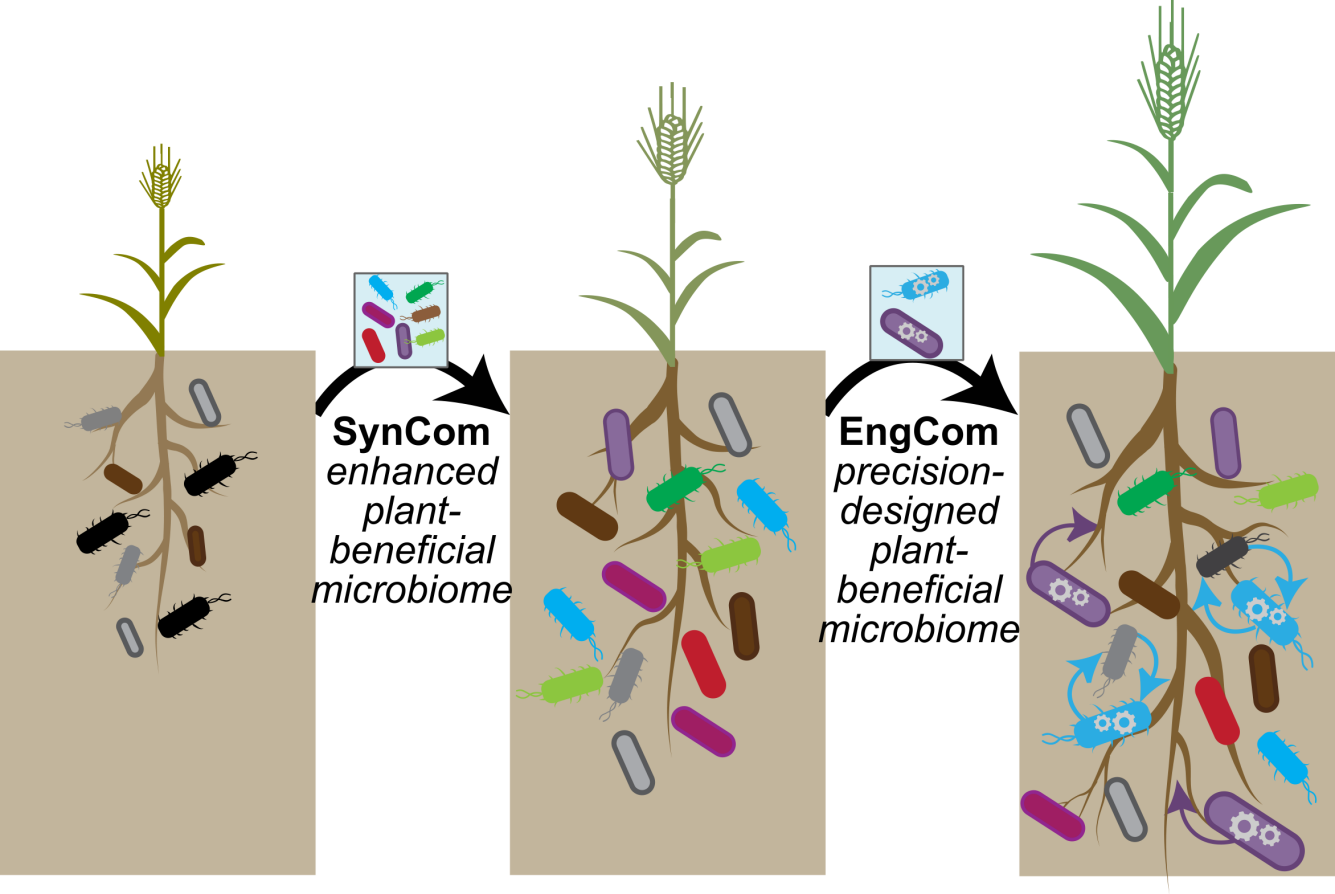
Conceptual transition from native soil microbiomes to SynComs and EngComs. Native soil microbiomes contain diverse micro-organisms that influence plant growth and health. The introduction of SynComs, assembled from selected microbial strains with beneficial traits, can enhance specific plant-beneficial functions such as nutrient acquisition. EngComs further extend these capabilities by incorporating genetically engineered strains ranging from trait-enhancing modifications to programmable functions, including biosensing and controlled interactions with the host plant. EngComs can be deployed either as engineered consortia alone or in combination with SynComs to further improve plant performance and resilience.

In this context, SynComs hold significant promise as next-generation bioinoculants and biocontrol agents. Unlike classical single-strain inoculants, SynComs can be designed to enhance colonization, functional redundancy and resilience in competitive and fluctuating field environments (Fig. 1). Recent advances in microbiome ecology have already enabled the development of ‘second-generation’ bioinoculants that are SynCom-based, including engineered communities [3], pointing towards a more predictive and durable use of microbial consortia in agriculture.

## Engineering microbiomes: from Syncoms to Engcoms

With the growing understanding of rhizosphere ecology and the rapid expansion of synthetic biology tools, it is now possible to move beyond SynComs towards EngComs (Fig. 1). We define EngComs as microbial communities composed of genetically engineered bacteria with wild-type bacteria (either natural microbiome or SynComs). Engineered strains are defined as strains with enhanced functions introduced through intentional genetic modification. They may range from simple trait-enhancing modifications, such as metabolic rewiring or targeted gene deletions, to sophisticated synthetic genetic circuits enabling sensing, communication, information processing, memory or conditional responses. In this review, we focus primarily on the latter, as programmable functions offer new opportunities to coordinate microbial behaviours and interactions within complex rhizosphere communities. These engineered strains can be designed to improve beneficial properties, such as: enhance plant-beneficial traits, promote stable colonization of the host by the community or increase the stability of the microbiome [48]. Advances in SynCom design, engineering biology, predictive tools and our understanding of plant-associated microbiomes are laying the foundations for the rational engineering of communities that function in complex and natural environments, where plants and endogenous microbial communities are already present. SynComs are designed to reduce community complexity while preserving key ecological functions; in contrast, EngComs introduce programmable functions into microbial communities, allowing targeted modulation of community behaviour and host interactions. Current efforts in engineering synthetic bacterial consortia have mainly focused on interbacterial communication, metabolic cross-feeding and engineered growth dependencies. Extending these principles offers the opportunity to rationally design microbial communities that operate robustly within native microbiomes and deliver enhanced benefits to the plant host in variable environments. Potential applications of EngComs and progress towards their development are described below.

### Engineering cell-to-cell communication

**Fig. 2.**
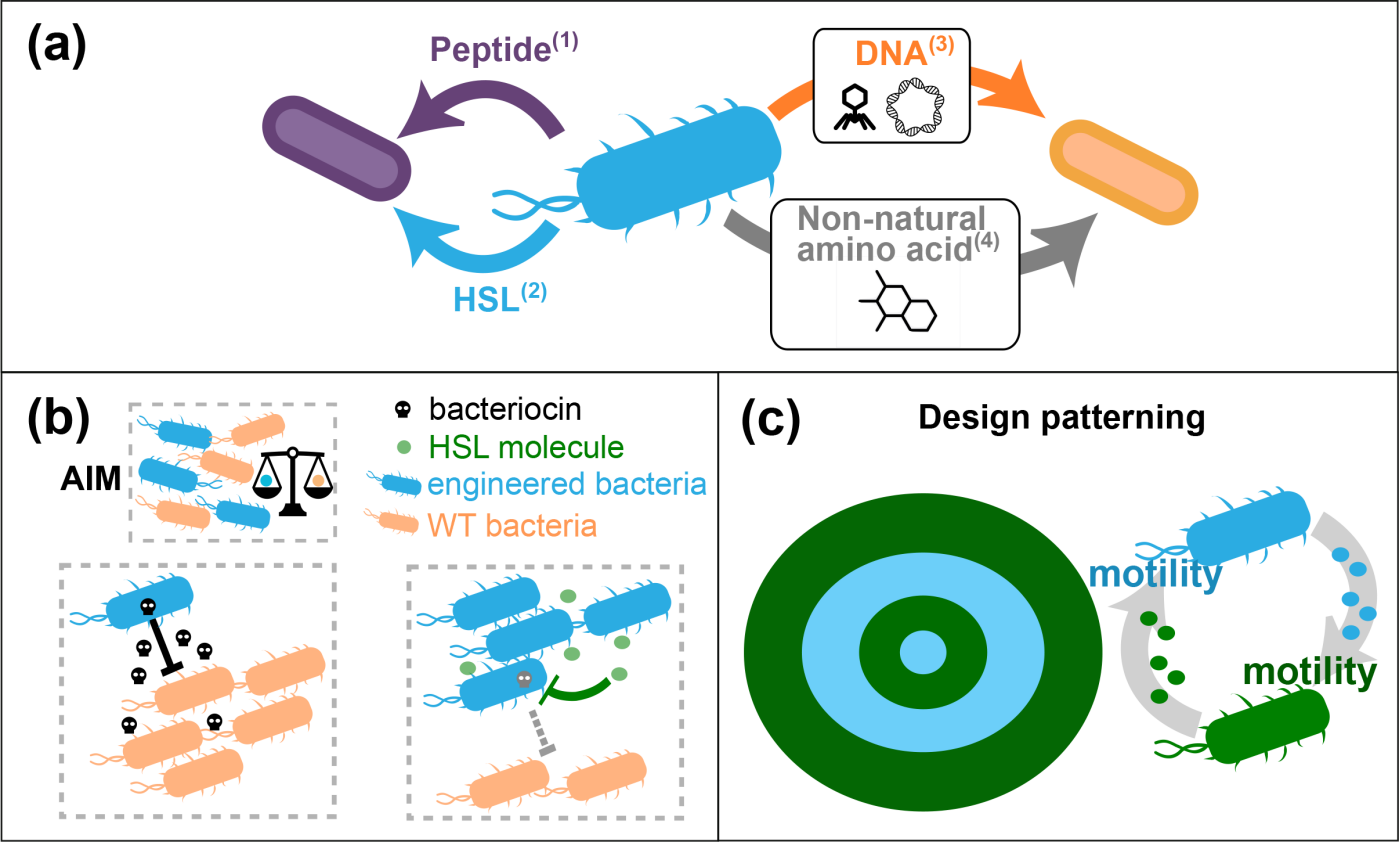
Engineering stable bacteria consortia by engineering communication and interdependence. (**a**) Cross-phyla communication via natural or non-natural signals. Examples of natural signals (left) include: (1) engineering of peptide production in a Gram-negative bacterium (in blue) to be detected by a Gram-positive bacterium (in purple) through its endogenous quorum-sensing pathway [55]; and (2) engineering in a Gram-positive bacterium to detect homoserine lactone (HSL) produced by a Gram-negative bacterium [56]. Examples of communication using non-natural signals (right) include: (3) using DNA via phage [59, 60] or via conjugation [61] or (4) using non-natural amino acid [61]. (**b**) Engineered toxin-mediated population control to stabilize a two-strain community by engineering a single strain to produce a toxin (bacteriocin) that reduces the growth of a competing WT bacterium, and this production is repressed by a quorum-sensing molecule, HSL, which is also produced by the engineered strain [64]. (**c**) Engineered cooperative ring patterns in *E. coli* consortia, by coupling quorum-sensing systems to the motility regulator CheZ, enabling density-dependent coordination of movement [69].

One approach to engineering microbial consortia is to control cell-to-cell communication as a first step towards establishing intercellular dependencies (Fig. 2A). In natural systems, quorum-sensing pathways constitute the primary means of communication within microbial populations. In Gram-negative bacteria, quorum sensing is predominantly mediated by acyl-homoserine lactones (AHLs), whereas Gram-positive bacteria typically rely on secreted oligopeptide signals. These quorum-sensing pathways have been engineered in both Gram-negative and Gram-positive bacteria. A wide range of orthogonal AHL-based quorum-sensing pathways have been implemented in *Escherichia coli* to construct gene regulatory networks and engineer intercellular communication [49, 50]. To increase the range of orthogonal signalling pathways in *E. coli*, peptide quorum systems such as Agr (from *Staphylococcus aureus*), PrgX (from *Enterococcus faecalis) [51]* and Com (from *Bacillus subtilis*) have been implemented in *E. coli*, validating the utility of Gram-positive peptide signalling components as modular genetic parts for communication networks [52]. While most engineering biology efforts have been conducted in *E. coli*, various quorum-sensing pathways have also been implemented in Gram-positive bacteria. For example, the *S. aureus* Agr quorum-sensing pathway was reconstituted in *Bacillus megaterium*, where sender cells produced autoinducing peptides and receiver cells activated gene expression in response [53]. The Gram-negative AHL quorum-sensing pathway has also been engineered in *B. subtilis* using the AHL-Lux complex to autonomously control the expression of a metabolic pathway limiting overproduction [54].

Cross-phyla communication has also been engineered. For example, Marchand and Collins [55] engineered communication from *E. coli* to *B. megaterium* by enabling *E. coli* to produce and secrete a peptide signal that could be sensed by the Gram-positive recipient (Fig. 2a). Multiple AHL-based quorum-sensing systems have also been successfully implemented in *B. subtilis [56]* (Fig. 2a). While not tested, this can enable the engineering of Gram-positive bacteria detecting Gram-negative communication channels. Together, these advances demonstrate that bidirectional communication between Gram-positive and Gram-negative bacteria can be synthetically engineered.

Recent progress now enables the construction of increasingly complex and multichannel communication networks across diverse bacterial taxa. A key remaining challenge, particularly in the context of EngComs operating in natural microbiomes, is the development of signalling systems that are orthogonal to endogenous quorum-sensing pathways. In this context, orthogonal signals are those that are neither produced, used, nor detected by the native microbial community. Achieving signal orthogonality is essential to minimize unintended crosstalk with native microbial communication networks and to ensure predictable, programmable behaviour of engineered communities *in situ*. One example of synthetic communication channels is the production of unnatural amino acids [57] (Fig. 2A). Numerous unnatural amino acids biosynthetic pathways [58] have now been engineered allowing orthogonal communication channels. Another alternative is the use of DNA-based messaging which can be delivered via bacteriophages (T7 RNA polymerase as message [59] and sgRNA [60]) or via targeted conjugation systems [61] (Fig. 2A). Together, these advances in engineering cell–cell communication not only improve our ability to coordinate microbial behaviours but also open the door to the design of more complex social interactions within microbial communities.

### Engineering social interactions

To engineer stable bacterial communities, social interactions may be manipulated to regulate the competition between strains and prevent the loss of consortia members over time. Two main strategies for tweaking social interactions are commonly employed – negative and positive – that can also be combined to create bidirectional relationships [48, 62, 63]. In negative interactions, a strain produces a toxin, as an example, that limits the growth of others, whereas in positive interactions, a strain secretes a molecule essential for the survival or growth of its partners.

An example of negative social interaction is the engineering of amensalism and competitive exclusion to stabilize a two-strain community (Fig. 2b). In this context, amensalism refers to a unidirectional interaction in which one strain inhibits the growth of another without being affected, while competitive exclusion describes the suppression of one population by another under shared conditions. Fedorec and colleagues [64] engineered an *E. coli* strain producing a toxin (bacteriocin, CvaC) to reduce the growth of a competing wild-type strain. To maintain population balance, the engineered strain employs quorum sensing to repress toxin production when its own density becomes too high. Notably, this system achieves stable two-strain consortia through the engineering of a single strain, making this approach particularly attractive for establishing engineered populations within complex natural microbiomes.

One example of positive social interaction is the use of unnatural amino acids to engineer obligate commensal relationships [57]. Here, the obligate commensal growth is dependent on the integration of a non-standard amino acid, which is produced by another engineered bacteria strain. This auxotrophy is engineered by adding a stop codon (TAG) at the start of an essential gene (adenylate kinase), only expressed in the presence of a non-standard amino acid (OMeTyr) produced by the partner bacteria. The interest of this system is that it is orthogonal to endogenous signals present in soils. While the system functioned effectively within a simplified maize root consortium composed of four strains (SynCom) in sterile media conditions, the impact of the two engineered bacteria on the four strains of the SynCom remains to be evaluated.

Building upon these foundational strategies, Kong and colleagues [65] demonstrated that a minimal genetic toolkit can generate the full spectrum of microbial social interaction types. Using just two antimicrobial peptides – nisin and lactococcin A (LcnA) – they engineered six distinct *E. coli* strains exhibiting commensalism, amensalism, neutralism, cooperation, competition and predation. To do this, they implemented the nisin and/or lactococcin A (*lcnA*) biosynthesis gene clusters in those strains. Both nisin and LcnA are used as antimicrobial molecules for negative interactions. Additionally, nisin is used as a signalling molecule to engineer mutualism. Specifically, one strain produces nisin, which induces expression of an essential gene (controlled by a nisin-responsive TetR promoter) in a partner strain, thereby coupling the survival of the second strain to the presence of the first. This paper shows that with a reduced set of components a wide variety of bacterial consortia can be built. However, those systems have been only tested in sterile laboratory conditions, and several of them require antibiotic supplementation (tetracycline) to maintain strain selection.

These examples show the potential of engineering bacterial consortia. The field will soon be mature enough to apply these strategies to engineered communities in non-sterile conditions, for example in the rhizosphere.

### Genetic circuits in engineered consortia

Multicellular organisms achieve functional complexity through cellular differentiation, whereby specialized cell types perform distinct tasks that collectively sustain the organism. Similarly, unicellular organisms use division of labour with different strains or differentiated cells from a single strain working together to perform complex programmes. One example is lichens combining cyanobacteria and fungi to create multicellular-like organisms, or biofilm formation in *B. subtilis* where bacteria differentiate into two cell types [66–68].

Similar to these natural consortia, engineering biology aims at using division of labour and synthetic gene circuits to engineer complex microbial systems with programmable and multicellular-level functions. The engineering of cell–cell communication and social interactions, as discussed above, is the first step toward this objective in rhizosphere EngComs. A proof of concept for gene circuits in engineered consortia is the engineering of spatial design patterns [69, 70]. Curtatolo and colleagues engineered cooperative ring patterns in *E. coli* consortia by coupling quorum-sensing systems (LuxI/LuxR and LasI/LasR) to the motility regulator CheZ, enabling density-dependent coordination of movement [69] (Fig. 2C). This work represents an initial step toward the engineering of complex multicellular structures [71].

Together, these examples illustrate how synthetic gene circuits can be used to coordinate behaviours within microbial consortia. Extending these approaches to rhizosphere contexts will require moving beyond bacteria–bacteria interactions to incorporate the plant as an active partner, enabling the design of circuits that mediate controlled and dynamic communication between microbes and their host.

## Engineering plant–microbe communication

Plants and soil microbes engage in a chemically rich and dynamic dialogue that forms the basis of plant health, nutrient acquisition and stress resilience. Root exudates, including sugars, amino acids, organic acids and phytohormone precursors, influence microbial chemotaxis and quorum sensing [20]. These signals shape microbial community composition and activity, for example by promoting the suppression of pathogens. Recent studies have further revealed that specific exudates can structure microbial colonization patterns and selectively recruit beneficial microbes, as illustrated by localized glutamine leakage shaping root colonization [72], and heptadecanoic acid attracting protective microbial communities to rice roots [73]. In parallel, specific microbial chemical signals can activate plant immune pathways such as induced systemic resistance (ISR) [74]. However, despite notable cases of specificity, this natural chemical language is often diffuse, context-dependent and difficult to control across heterogeneous soil environments [25]. Engineering these native signalling plant–microbe pathways provides a foundation to rebuild them for programmable, robust and precise communication exchange.

**Fig. 3.**
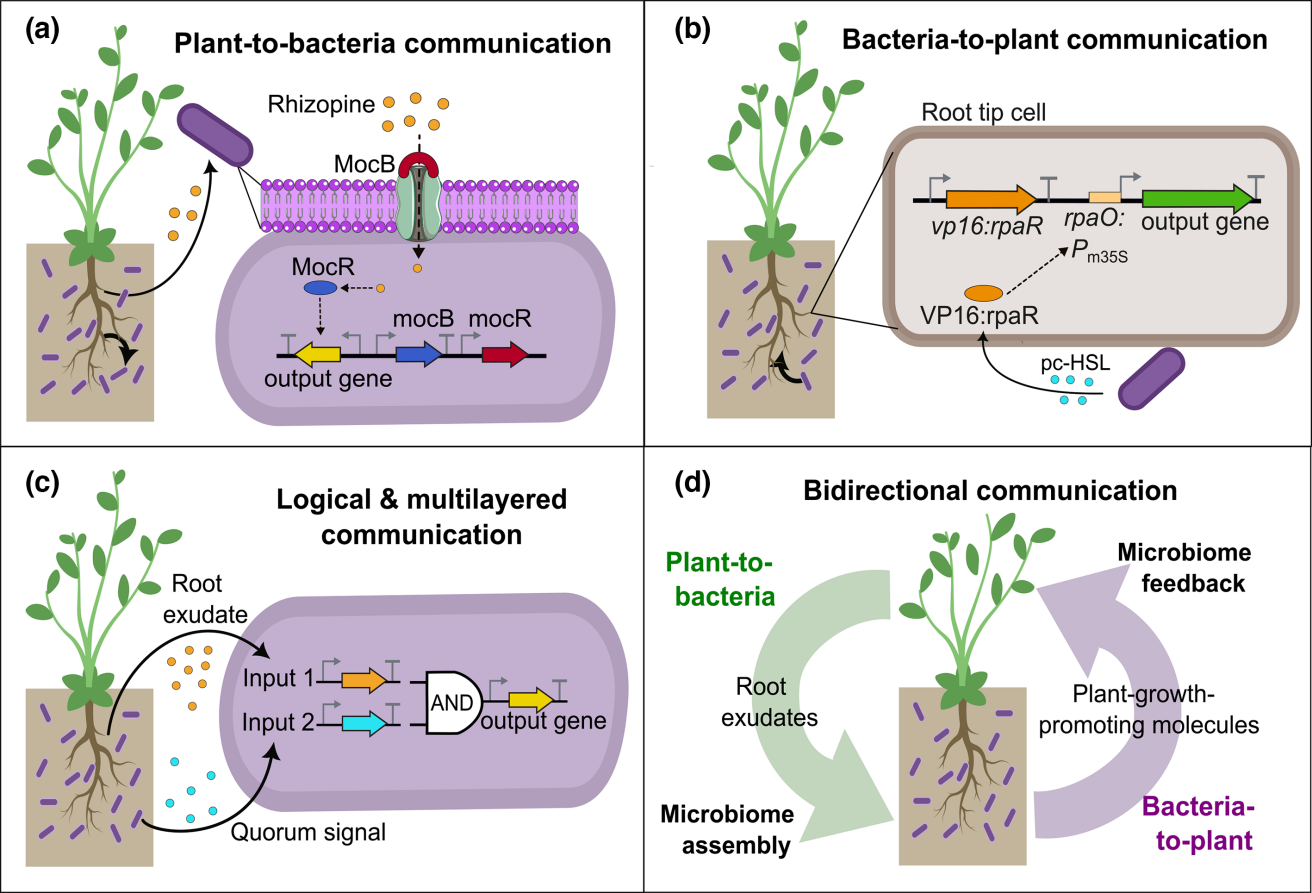
Engineered plant–microbe communication channels. (**a**) Plant-to-bacteria communication. Transgenic plants engineered to synthesize and secrete rhizopine enable specific activation of gene expression in rhizosphere-colonizing bacteria. The bacterial solute-binding protein MocB captures extracellular rhizopine and delivers it through a transporter complex for cellular import. Intracellular SI binds the transcription factor (TF) MocR, which activates expression from the PmocB promoter, enabling plant-dependent control of bacterial reporter genes or plant growth promoting (PGP) traits such as nitrogen fixation. (**b**) Bacteria-to-plant communication. Engineered bacteria synthesize the orthogonal quorum-sensing molecule p-coumaroyl-HSL (pC-HSL), which is perceived by plant roots engineered to express the chimeric receptor VP16:RpaR. Signal binding activates transcription from the RpaO:Pm35S synthetic promoter, enabling bacteria-to-plant signalling. (**c**) Logical and multilayered communication. Integration of multiple environmental inputs (root exudates and bacterial quorum-sensing molecules) through synthetic gene circuits acting as ‘AND gates’ enables context-dependent gene activation only when bacteria are root-associated and at sufficient cell density. (**d**) Bidirectional and feedback-based communication. Reciprocal signalling modules where plants recruit specific microbiome members through root exudates (green arrow) and bacteria modulate plant physiology through secreted plant-growth-promoting molecules (purple arrow), establishing feedback loops for microbiome assembly and function.

Engineering plant-to-microbe signalling enables plants to send precise molecular cues that selectively activate engineered traits in beneficial bacteria. Because natural exudates tend to be metabolically costly and promiscuous in their effects, recent approaches favour orthogonal small molecules that do not overlap with endogenous pathways. A notable example is the rhizopine-dependent system, initially designed by Geddes and colleagues [75] and further applied by Haskett and colleagues [76]. In this system, transgenic plants are engineered to synthesize rhizopine, a bacterial signalling molecule that *Sinorhizobium meliloti* naturally produces and senses via the MocR regulatory system [75]. When rhizopine-responsive biosensor plasmids are introduced into the rhizosphere-colonizing bacterium *Azorhizobium caulinodans*, plant-derived rhizopine activates heterologous gene expression *in situ* on root surfaces [76] (Fig. 3A). Moving beyond natural signalling molecules, Zhong and colleagues recently demonstrated plant-directed control of bacterial activity through genetic code expansion [77]. In this system, plants provide a non-natural amino acid required for bacterial protein synthesis, creating an orthogonal communication channel that allows host-dependent regulation of engineered bacterial functions. Such designs illustrate how plant-derived metabolites can serve as control signals for microbial performance. Beyond controlling microbial behaviour, engineered plant–microbe communication can also enable plants to act as living reporters of rhizosphere processes. Recently, Wang and colleagues developed ‘sentinel plants’ capable of converting rhizobacterial pC-HSL (homoserine lactone) production into GFP expression on the aboveground plant tissue [78]. This work expands the use of engineered communication channels to transform plants into sensors of rhizosphere function.

Conversely, microbes can be engineered to transmit chemically encoded information back to the plant. Boo and colleagues [79] created modular ‘sender’ circuits in *Pseudomonas putida* and *Klebsiella pneumoniae* that synthesize the orthogonal quorum-sensing molecule p-coumaroyl-HSL (pC-HSL) in response to diverse inputs (e.g. IPTG, aTc, arsenic) (Fig. 3B). Notably, pC-HSL is not endogenous to these chassis bacteria nor typically found in soil; it is a specialized signal produced naturally only by select bacteria such as *Rhodopseudomonas palustris*, making it an effectively orthogonal communication channel. pC-HSL diffuses across plant tissues and is detected by a synthetic ‘receiver’ module in engineered lines of *Arabidopsis* and potato, where it activates gene expression. Therefore, microbial production of these specific AHL-type signals provides a direct route to modulate plant transcriptional programmes. Such engineered microbe-to-plant channels enable bacteria in the rhizosphere to process environmental cues and forward precise, orthogonal signals into plant regulatory circuits.

Emerging multilayered design frameworks are beginning to transcend the limits of classic one-way communication by also considering the ‘scenario’ of the interaction. Van Donk and colleagues [80] exemplified this shift, by identifying orthogonal chemical channels and optimizing circuit dynamics, the team built a biosensor that integrates a salicylic-acid-responsive root-exudate detector with a LuxI/LuxR quorum-sensing module through a toehold-switch AND gate (Fig. 3C). This ensures gene activation only when bacteria are both root-associated and at sufficiently high density, demonstrating how multi-input logic and environmental context can produce circuits that remain reliable within real rhizosphere constraints. Following similar contextual approaches, and given that plant–microbiome interactions are inherently reciprocal, van Rensburg and colleagues [81] recently argued that single-direction signalling modules are unlikely to capture the actual dynamic interplay and therefore need to be integrated into coordinated, bidirectional architectures (Fig. 3D).

As engineering efforts progress from isolated circuits towards bidirectional, multilayered, context-aware networks, the field is converging on communication systems that are modular, predictable and ecologically grounded. Key next steps include embedding orthogonal signalling within natural rhizosphere processes, ensuring compatibility with plant and microbial fitness.

## Engineering rhizobacteria to sense, record and respond with functional outputs

Engineering rhizobacteria represents a powerful strategy to both monitor and enhance agricultural systems. Microbial biosensors enable the detection of biologically relevant signals directly in the rhizosphere. In parallel, engineered bacteria can be designed to promote plant growth by improving nutrient availability. Together, these approaches contribute to the development of more precise agricultural practices and offer promising avenues to increase crop productivity while reducing environmental impact.

### Biosensing circuits for monitoring soil health

Monitoring soil health remains difficult because soil is a highly complex and dynamic environment. Many key processes such as nutrient cycling, plant signalling and pathogen interactions are driven by transient chemical signals exchanged between microbes and plants. Traditional soil analyses mostly provide static measurements and often fail to capture these biological interactions in real time [82, 83]. Engineered microbial biosensors provide a promising alternative. By using sensing systems such as transcription factors (TFs) or riboswitches coupled to synthetic circuits, bacteria can be programmed to detect environmental cues. Rhizobacteria are particularly suitable for this purpose because they naturally colonize plant roots and are directly exposed to signals that reflect soil and plant health.

Engineered biosensors for diagnostics and environmental monitoring are organized around three essential circuit layers: a sensing module that captures chemical or biological signals, a signal-processing element that amplifies or filters the input, and an output system that generates a detectable readout [84]. A recurrent tool emerging across biosensor development is the use of serine integrases as processing modules capable of converting transient molecular events into permanent genomic records. Integrases recognize specific asymmetrical DNA sequences, known as attachment sites (attP and attB) and either invert or delete the DNA fragment between them, depending on their orientation [85]. This transforms transient chemical information into a stable genetic state; a critical advantage in soils where metabolite concentrations fluctuate rapidly.

**Fig. 4.**
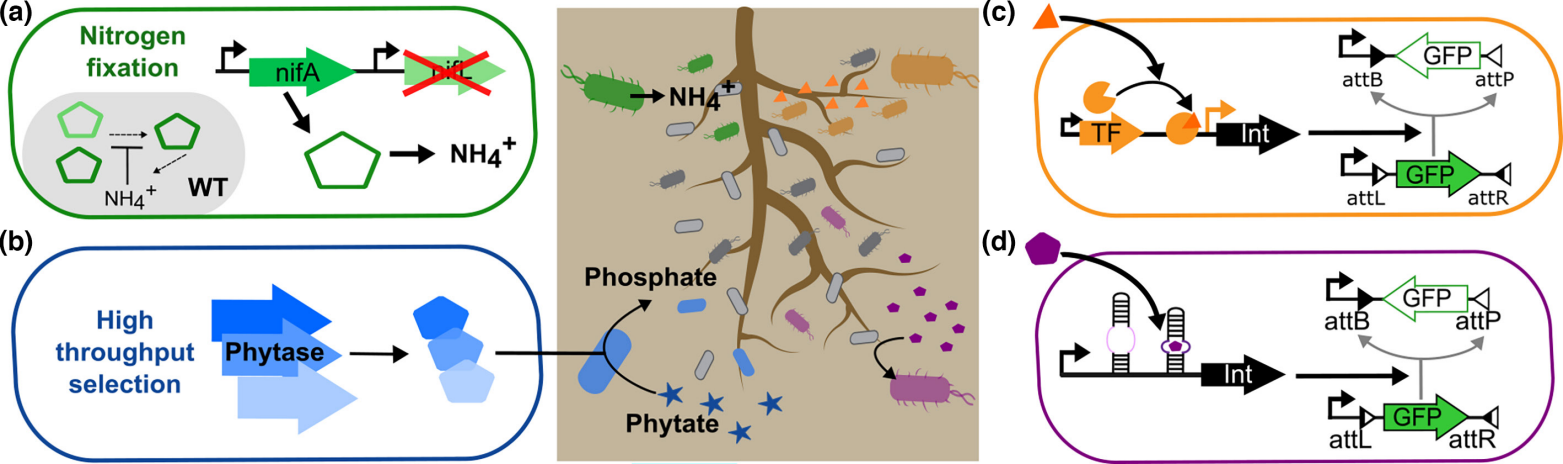
Examples of rhizobacteria engineering strategies for improving nutrient availability and environmental sensing in the rhizosphere. (**a**) Engineering of nitrogen fixation regulation in a free-living diazotroph [92]. In the wild-type system (grey), the NifL regulatory protein (light green hexagon) inhibits NifA (dark green hexagon), limiting nitrogen fixation in response to environmental nitrogen levels. In the engineered system, deletion of *nifL* and constitutive expression of *nifA* allow continuous activation of the nitrogen fixation pathway, resulting in increased ammonium production (NH_4_^+^). (**b**) Engineering bacteria for improved phosphate mobilization from organic sources [93]. Candidate bacterial strains expressing heterologous phytases (in blue) are screened through a high-throughput selection workflow. Engineered strains are shown converting phytate (organic phosphorus, blue stars) into soluble phosphate. (**c**) Design of a TF-based biosensor for detecting rhizosphere signals in *P. putida [91]*. A signal-responsive TF regulates expression of an integrase (Int) depending on the presence of a molecule of interest (orange triangle). Upon activation, the integrase catalyses recombination between attB and attP sites (triangles), resulting in inversion of a DNA cassette and switching reporter expression (GFP). (**d**) Riboswitch-based chemical sensing circuit in *B. subtilis [86]*. Binding of TNT (purple hexagon) to a specific riboswitch induces a conformational change that enables translation of an integrase (Int). Similar to panel (c), integrase-mediated recombination between att sites results in inversion of a DNA element and activation of a reporter gene.

Essington and colleagues [86] illustrate the potential of such genetic circuits with a biosensor for TNT (trinitrotoluene) (Fig. 4D). In their system, detection relies on an RNA aptamer located upstream of the integrase Int2 coding sequence [85]. In the absence of TNT, the aptamer adopts a structure that blocks protein synthesis. However, in the presence of TNT, ligand binding induces a conformation change allowing for the transcription of the protein. This purely structural mode of sensing bypasses the need for protein TFs and can discriminate between related nitroaromatic compounds after optimization [87]. Although this approach is powerful, it has several limitations. The range of molecules that can be detected by aptamers remains relatively limited due to constraints inherent to their structure. Indeed, for some molecules, it is highly challenging to design aptamers with high specificity due to their structure [88].

Alternatively, numerous biosensors based on TFs have been developed for molecules relevant to the rhizosphere and could be or have been applied to soil detection of molecules of interest [80, 89–91]. For example, Hansen and colleagues [91] developed a TF-based system in *P. putida* that detects biotic signals such as DAPG (2,4-acetylphloroglucinol), pyoluteorin, salicylate and anhydrotetracycline (Fig. 4C). The system relies on metabolite-responsive TFs that activate the expression of an integrase gene. These TFs (PhlF, PltZ, NahR and TetR) operate as ligand-dependent repressors. In the absence of their cognate metabolite, they block transcription, whereas ligand binding induces a conformational change that relieves repression, thereby turning on the expression of the integrase, Int12. Int12 inverts a barcode acting as a permanent record of signal exposure. Importantly, the authors tested system functionality in SESOM (soil-extracted solubilized organic and inorganic matter), a sterile soil-like composition liquid medium, further supporting the robustness of the circuit and reinforcing the potential of *P. putida* as a rhizosphere chassis. However, this system has not yet been tested in real soil environments or in complex microbial communities.

### Plant growth-promoting treatments for crop resilience

Engineered bacteria can be used not only for sensing but for actively improving plant growth. Many plant-associated bacteria can be modified to enhance nutrient availability in the rhizosphere, for example by increasing nitrogen fixation [92], mobilizing phosphorus through phosphatase or phytase activity [93] or improving iron acquisition via siderophore production [94]. Engineered microbes can also influence plant development through the production or modulation of phytohormones [84] such as auxins, cytokinins or ethylene. In this section, the focus will be on strategies that enhance nitrogen and phosphorus availability, which are two major limiting nutrients in agricultural soils, although many additional approaches for engineering beneficial plant–microbe interactions are currently being explored.

Nitrogen availability remains one of the most limiting factors in global agriculture and most of the nitrogen bioavailable for plants is either in the form of ammonium or nitrate. Wen and colleagues [92] addressed this challenge by constructing the first nitrogen-fixing bacteria capable of functioning in fertilized soils, which is known to reduce the efficiency of microbial nitrogen fixation [95] (Fig. 4A). They engineered *Klebsiella variicola* by deleting *nifL*, encoding the inhibitory sensor protein that blocks *nifA* activation when ammonium is present, and replacing the native regulatory region of *nifA* with a constitutive promoter. This rewiring decouples nitrogenase expression from external ammonium availability. The resulting strain, Kv137-1036, maintained high nitrogenase activity even under high ammonium concentrations, releasing substantial amounts of bioavailable ammonium into the rhizosphere. In greenhouse and multiyear field trials, the engineered strain colonized maize roots effectively and consistently increased nitrogen fixation and yield, demonstrating that targeted edits to gene regulatory elements can overcome the long-standing repression barrier in diazotroph deployment. These efforts ultimately led to the commercialization of this strain in the United States.

Phosphorus is another major constraint on plant productivity, as most of it is locked in phytate (IP6) and therefore unavailable to plants. Shulse and colleagues [93] addressed this bottleneck by mining thousands of genomes and metagenomes to identify more than 2,500 candidate phytase genes spanning the major phytase families (HAPs: Histidine Acid Phosphatase, CPs: Cysteine Phosphatase, and BPPs: Beta-Propeller Phytase), which transform phytate into phosphate (Fig. 4B). Using the BOOST (Build-optimization software tools) DNA-optimization pipeline and high-throughput methods, they refactored a phylogenetically diverse subset of 82 enzymes for high-level expression in Proteobacteria, standardizing codon usage, RBS (ribosome binding site) context and secretion signals. These optimized genes were stably integrated into the genomes of three root-associated hosts, *Pseudomonas simiae*, *Ralstonia* sp. and *P. putida*. When screened on phytate as the sole phosphorus source, dozens of these strains efficiently released orthophosphate in liquid LB (Luria–Bertani). Several of the top producers also promoted *Arabidopsis* growth in laboratory settings, demonstrating functional activity *in planta* in controlled environments.

## Genetic toolkit and engineering methods for rhizobacteria

**Fig. 5.**
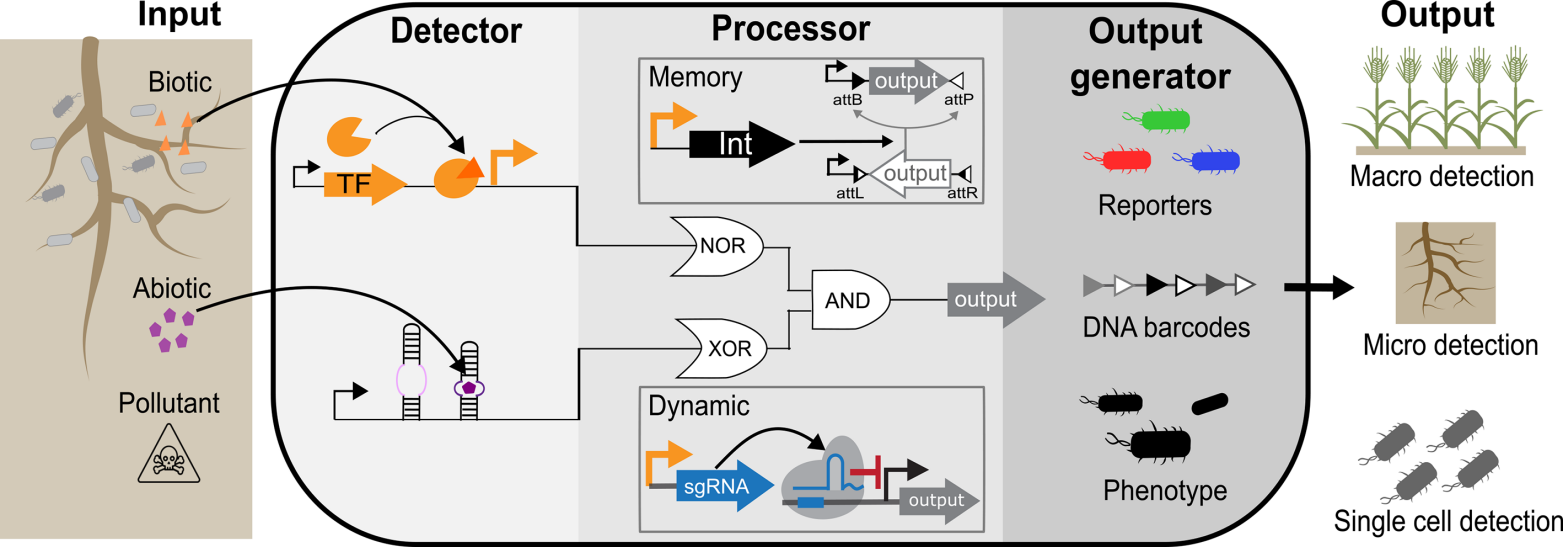
Modular architecture of rhizobacteria engineering. Rhizobacteria encounter diverse biotic and abiotic (naturally occurring or pollutant) signals present in the soil. These signals are recognized by two main types of sensor systems, TF-based sensors (activators or repressors) and riboswitches, which regulate translation upon ligand binding. The detection unit controls a processing layer, which integrates signals and performs logic operations. This layer can make use of several methods such as site-specific integrases (top box), CRISPR-based regulation (bottom box) or other synthetic circuits to implement logic gates (AND, OR, NOT…), enhancing specificity and enabling scalable multiplex sensing. Processed signals are transmitted to an output module, which can produce reporter proteins (e.g. fluorescent, hyperspectral), DNA barcodes for environmental memory or phenotypic changes to improve plant growth. Finally, these outputs can be observed across multiple spatial scales, from macro detection (e.g. of field using drones), to micro detection (e.g. microscopy using fluorescent protein), to single-cell detection using sequencing.

Constructing functional genetic circuits in rhizobacteria requires integrating multiple biological layers that can be deconstructed into three core modules: (i) a sensing module that detects specific environmental or plant-derived signals, (ii) a processing module that integrates and regulates the sensed information and (iii) an output module that executes a measurable or functional response (Fig. 5). Together, these three modules convert input signals into outputs that correspond to the information sought or the desired functional outcome.

Inputs represent the physical or chemical cues that the bacterium is engineered to detect, such as abiotic (e.g. pollutants, metals), plant-derived (sugars, organic acids, phenolics) or microbial sources (antibiotics, siderophores, quorum signals, specialized metabolites). In a synthetic signalling circuit, the sensing module will mediate the detection of these endogenous or exogenous inputs. TF/inducible promoter systems remain the most widely used sensing system due to their quantitative properties, dynamic range and diversity [84]. In this system, a TF binds to the promoter and either activates or represses it. Much work has been done to identify TF-promoter partners and optimize TF promoter [80, 89–91]. Although TF-promoter systems dominate, other sensing strategies exist. RNA-based sensors, particularly riboswitches, provide compact and low-burden alternatives by modulating translation initiation or transcription termination through ligand-dependent folding transitions, such as in the TNT biosensor [86] (Fig. 3D), where ligand-induced RNA conformational changes modulate ribosome accessibility. Some studies have also explored the use of two-component systems as sensing modules [96]. These systems consist of a membrane-bound histidine kinase that detects an external signal and a response regulator that controls gene expression. Two-component systems are naturally widespread in bacteria and can respond to diverse environmental cues such as nutrients, stress signals or host-derived compounds. However, compared with TF-based sensors, they are generally more difficult to repurpose into synthetic circuits. Their activity often depends on species-specific protein interactions, phosphorylation dynamics and membrane context, which can limit their portability between bacterial hosts [96].

The processing layer transforms sensor signals into stable, amplified or logically structured signals. Many synthetic biology circuits rely on one-input transcriptional regulation per sensing module, where networks of activators and repressors can create logic gates such as AND, OR or NOT [97] and help filter noise or strengthen weak signals by modifying the affinity of the sensing module or of the logic gate [98]. Additionally, a particularly powerful class of processing tools with memory are site-specific recombinases, such as serine integrases. By recombining asymmetric attP/attB sites, integrases mediate irreversible DNA inversions/deletion when the sites are oriented antiparallel/parallel, respectively. This allows signals to be permanently recorded even after the original stimulus disappears. More complex logic circuits can be implemented using multiple integrases, each responding to one signal and placing integrase sites around promoters/genes. Depending on the respective position of the sites, different logic circuits are implemented. Processing can also occur post-transcriptionally through RNA-based regulators such as riboregulators, small RNAs or toehold switches that modulate translation. More recently, programmable systems based on CRISPR-based activation (CRISPRa) and interference (CRISPRi) have been used to construct complex regulatory circuits [99]. In addition, protein-level mechanisms provide further control, including phosphorylation cascades, protease switches or split proteins that only become functional when multiple inputs are present [100].

The output layer of the circuit provides either a detectable reporter signal or the production of a functional molecule of interest. For detection, fluorescent and bioluminescent reporters remain standard for microscopy and microfluidics. Newly developed hyperspectral reporters allow long-distance and wide-area detection through spectral unmixing against soil autofluorescence [101]. Alternatively, integrase-based DNA barcodes provide an increasingly useful non-destructive output modality. The recombination state of the DNA cassette can be quantified across bacterial populations using qPCR or sequencing [91], enabling durable readouts even when fluorescent signals fail in opaque soil environments. In addition, circuit output can be the production of a protein or molecule of interest that has a functional benefit (e.g. pythase), thereby enabling environmental or commercial applications.

Constructing these circuits depends on robust modular DNA assembly frameworks that support high-throughput testing. Type IIS restriction enzyme-based cloning strategies, such as Golden Gate assembly, enable modular, standardized construction of increasingly complex synthetic genetic circuits. Several specialized Golden Gate-based toolkits enable the construction of complex, broad-host-range circuits suitable for rhizobacteria. SevaBrick merges the standardized SEVA vector layout [102] with MoClo/Phytobrick syntax, allowing plug-and-play exchange of origins of replication, antibiotic resistance cassettes and expression modules through Golden Gate reactions. This modularity is particularly advantageous for multiplasmid systems in which sensing, processing and reporting modules are distributed across plasmids to reduce burden and avoid crosstalk. The BEVA system (Bacterial Expression Vector Architecture) [103], derived from the SEVA toolkit, offers a curated library of well-characterized promoters, RBS sequences and fusion tags within a Golden Gate-compatible architecture optimized for Gram-negative bacteria. For Gram-positive bacteria, the SubtiToolKit [104] implements similar hierarchical design principles providing collections of diverse genetic parts, functionally validated in *B. subtilis* and *Geobacillus*. Finally, expanding the repertoire of usable genetic parts increasingly relies on computational DNA design frameworks. These tools enable the rapid generation and optimization of large libraries of sequences by automating tasks such as codon optimization, G+C-content balancing, removal of inhibitory motifs and elimination of internal type IIS restriction sites. By systematically domesticating sequences while preserving function, such approaches make it easier to adapt biological components for standardized cloning strategies and broaden the range of parts available for synthetic biology [105].

Selecting an appropriate bacterial chassis is a critical step for deploying engineered systems in the rhizosphere. Different bacterial genera offer distinct advantages depending on ecological fitness, genetic tractability and compatibility with the intended circuit design. *Pseudomonas* spp., particularly *P. putida* and *P. fluorescens*, are widely used due to their strong root colonization capacity, metabolic versatility and tolerance to oxidative stress, osmotic fluctuations and phenolic compounds [106]. Their genetic toolkits are relatively well developed, facilitating modular cloning and genomic integration. However, aspects of their intrinsic physiology can interfere with circuit performance. For example, efflux pumps in *P. putida* can actively export plant-derived flavonoids, potentially limiting intracellular detection in biosensor designs [107]. *Bacillus* spp. provide a complementary platform. Their major advantage lies in their ability to form endospores, enabling environmental persistence, long-term storage and ease of field application. They are also naturally associated with plant roots and widely used in agricultural bioproducts [108]. However, compared with *Pseudomonas*, their regulatory networks are less well-characterized and their synthetic biology toolkits remain less standardized, which can hinder the construction of highly complex circuits. Beyond these established chassis, non-model rhizosphere bacteria such as *Klebsiella* spp. may offer niche-specific advantages [92]. Emerging initiatives such as the non-profit organization Cultivarium [109] together with recent advances in scalable genetic toolkits and engineering strategies for non-model and previously undomesticated micro-organisms [110, 111], aim to expand synthetic biology capabilities beyond a limited set of traditional chassis organisms. These efforts seek to overcome barriers in genetic tractability and enable the integration of a broader diversity of ecologically relevant species into engineered microbial systems. Ultimately, choosing a microbial chassis involves a three-way trade-off between ecological fitness, circuit assembly and compatibility with the specific sensing or metabolic outputs intended.

## Current limitations in engineering rhizobacteria for plant health

Despite major advances in synthetic biology and plant–microbe research, several technological challenges still limit the rational engineering and deployment of rhizobacteria in realistic soil environments. Key bottlenecks include the difficulty of monitoring engineered microbes *in situ*, the limited ecological realism of current experimental platforms, the lack of robust genetic orthogonality in complex microbiomes and unresolved biocontainment constraints, as well as regulatory and ethical considerations that will ultimately determine field deployment.

### Imaging and monitoring engineered microbes in the soil

A major technical limitation is the ability to directly visualize and track engineered microbes in opaque, heterogeneous soils. As a result, most studies rely on indirect or simplified monitoring approaches (reviewed in [112]). In controlled laboratory settings, optical reporter-based assays using bioluminescent or fluorescent proteins enable non-invasive monitoring of gene expression and microbial activity [113]. However, their application in soil is constrained by strong signal attenuation and limited depth penetration, typically requiring sample recovery or the use of transparent or semi-artificial systems such as hydrogels [114, 115], microfluidic rhizosphere-on-a-chip platforms [116] or superficial root-associated zones [117]. While hydrogel-based soils enable high-resolution optical imaging of root–microbe interactions, they simplify soil chemistry and pore architecture, whereas microfluidic systems trade ecological realism for experimental control and reproducibility.

Beyond optical approaches, X-ray micro-computed tomography (microCT) enables non-invasive three-dimensional visualization of soil structure, pore networks and root architecture in controlled settings, but cannot directly resolve microbial populations or engineered gene expression [118]. In field conditions, such approaches become inherently invasive, as they require soil sampling or the insertion of detection systems. More recently, multimodal sensing platforms that integrate X-ray imaging with metabolite profiling, microbial analysis and computational mapping have begun to provide a more holistic view of rhizosphere processes, although their application also remains limited by soil opacity, technical complexity and scalability constraints [119].

Several emerging approaches aim to overcome these depth and resolution limitations. Hyperspectral imaging, combined with engineered bacterial pigment reporters, such as bacteriochlorophylls and carotenoids with distinct near-infrared spectral signatures, enables non-invasive, wide-area detection of bacterial gene expression at centimetre-scale depths in opaque soils [101]. Similarly, thermoacoustic imaging [120] and gas-vesicle-based acoustic reporters [121] hold promise for deep, non-invasive monitoring of engineered microbes in real soil environments, although these technologies remain at an early experimental stage and require substantial optimization for agronomic deployment.

Finally, multimodal and multiscale phenotyping platforms that integrate automation with dedicated image-analysis pipelines, such as the Growth and Luminescence Observatory for Roots, are under development [122]. These systems aim to combine imaging, sensing and modelling to capture microbial dynamics across scales, but remain fundamentally limited by soil opacity and system complexity.

Beyond reporter systems, genetic barcoding strategies are emerging as powerful tools for tracking individual strains within complex microbial communities, enabling high-resolution monitoring of population dynamics, strain persistence and community assembly processes in both SynComs and EngComs [123, 124].

### Mimicking natural soil environments and ensuring circuit robustness in complex microbiomes

To achieve experimental tractability, engineered rhizobacteria are typically tested in simplified conditions: sterile substrates [75], reduced SynComs or microfluidic devices [125, 126]. While these systems enable reproducibility and fine control over environmental variables, they fail to capture the taxonomic diversity, spatial heterogeneity and competitive interactions characteristic of real soils. As a result, ensuring that engineered circuits function predictably within complex microbiomes remains a major challenge.

Orthogonal signalling pathways may be disrupted by native metabolic noise, cross-feeding interactions [127], degradation of signalling molecules or unintended activation of endogenous regulatory systems [48]. Soil microbial communities also engage in widespread horizontal gene transfer [128], creating risks of circuit loss, modification or escape into non-target taxa. Designing circuits that remain resilient to environmental variability (pH shifts, osmotic fluctuations, nutrient scarcity, oxygen gradients) therefore requires integrating synthetic biology with ecological and biophysical modelling. Although mathematical modelling can be coupled with empirical SynCom experiments [129], this integration is still maturing, and current models struggle to capture the emergent, multiscale behaviours that define rhizosphere ecosystems. Efforts such as the synMADE framework [130] have begun to address these limitations by proposing cross-ecosystem standards for constructing and benchmarking synthetic microbial communities. Such standardization may become essential for systematically evaluating engineered strains across diverse soil types, plant hosts and environmental conditions.

### Biocontainment considerations

The safe deployment of engineered microbes in open environments demands robust, multilevel biocontainment [131, 132]. However, recent works underscore the fragility of many existing approaches [133]. Environmental fluctuations [134] including temperature shifts, osmolarity changes and nutrient limitation can destabilize auxotrophies, toxin–antitoxin modules and kill switches. Additionally, mutation, metabolic rewiring and horizontal gene transfer remain major failure modes.

A recent review on environmental release [135] emphasizes rapidly tightening regulatory expectations, with agencies increasingly requiring redundant, orthogonal fail-safe mechanisms. One emerging strategy involves engineering dependency on non-natural metabolites. For example, Pigula and colleagues [136] engineered *P. aeruginosa* to require the unnatural amino acid *p*-benzoyl-l-phenylalanine for growth by incorporating it into the essential DNA replication protein DnaN. This design creates a stringent auxotrophy with extremely low escape frequency (<10⁻¹¹), effectively preventing replication in environments lacking the synthetic amino acid. Parallel work in mammalian systems has introduced advanced strategies such as STALEMATE [137], which entangles essential genetic sequences to buffer circuits against mutation; adapting such concepts to soil bacteria may provide new avenues for robust containment. Antibiotic-free plasmid systems [138] help reduce the ecological risks associated with antibiotic-resistance spread, but plasmids remain unstable in soil environments. As a result, genomic integration is often preferred. Marker-free genome integration is routinely achieved through site-specific recombination systems such as Flp/FRT or Cre/loxP [139], where antibiotic resistance cassettes flanked by recognition sites are excised after integration by expressing the corresponding recombinase. Alternatively, counterselection strategies using SacB (levansucrase, which confers sucrose sensitivity) [140] enable seamless deletion of selection markers without leaving residual scars, allowing repeated use of the same antibiotic resistance gene for multiple genomic modifications [141].

### Regulatory landscapes, ethical considerations, and future prospects

Regulatory frameworks for engineered micro-organisms vary widely across jurisdictions and often lag behind current microbial engineering capabilities [142]. The United States has historically adopted a relatively permissive stance, from early EPA-supervised releases of engineered *Pseudomonas* strains to recent multistate trials of nitrogen-fixing microbes. Also, they are currently updating their ‘Coordinated Framework’ with new guidance tools for developers. In contrast, authorizing even small-scale field trials in the European Union remains exceptionally challenging, as Member States retain veto power. While proposed updates to Regulation (EU) 2017/625 aim to accelerate the approval of plants developed using new genetic technologies – partially aligning with the UK Precision Breeding Act (2023) [143] – these changes do not extend to the environmental release of genetically modified micro-organisms, although they may facilitate indirect soil microbiome modulation via edited plants. This asymmetry contrasts with scientific practice, where microbial solutions often precede plant engineering due to shorter development cycles.

Approval timelines for field trials often span several years, creating a regulatory ‘Catch-22’: while empirical evidence of safety and ecological performance is required, restrictions on environmental testing limit the ability to generate such data. For example, developers of hyperspectral reporter strains [101] have reported spending several years navigating regulatory landscapes and assessing potential risks and benefits before any environmental deployment could be considered.

Ethical considerations are also gaining prominence. Beyond biosafety, recent analyses have highlighted the importance of distributive justice, ecological stewardship, and long-term responsibility when deploying engineered organisms into shared environments [142]. Public acceptance will depend on clear agronomic value, transparency, and early engagement with farmers, communities, and policymakers. In this context, terminology may also influence perception. Very recently, several soil microbiome researchers have proposed replacing the term ‘synthetic microbial communities’ with ‘defined microbial communities’ for practical applications [144]. Concepts such as *soil probiotics* or *microbiome transplantation* may provide a more intuitive framework for communicating the introduction of beneficial microbial consortia, analogous to the use of probiotics to support human gut health. At the same time, ensuring that microbial innovations benefit low-resource farming systems will require open-science initiatives and equitable licensing frameworks.

Ultimately, responsible deployment of engineered rhizobacteria will require integrating ecological modelling, evolutionary foresight, coherent regulatory pathways, and inclusive stakeholder dialogue. Standardized monitoring of persistence, dispersal and horizontal gene transfer will be essential to evaluate both efficacy and environmental impact, enabling microbial interventions that are effective as well as socially responsible.

## Discussion

The engineering of rhizobacteria is transitioning from descriptive microbiome studies toward a more predictive and design-driven discipline. Across this review, we have highlighted how advances in SynCom assembly and engineering biology are converging to enable the development of EngComs and increasingly precise control over microbial behaviours. However, translating these designs from controlled systems to complex soil environments remains a major challenge, requiring improved robustness, orthogonality, and integration with ecological and biophysical frameworks.

Importantly, most current efforts focus on bacterial systems. However, the rhizosphere is shaped by a much broader diversity of organisms, including fungi and archaea, which play key roles in nutrient cycling, plant health and ecosystem stability. Although these organisms remain challenging to manipulate, expanding engineering approaches to encompass multikingdom interactions will be essential to fully capture and harness rhizosphere functionality. Progress in microbial cultivation, genome editing, and systems-level understanding is likely to enable the next generation of SynComs and EngComs that incorporate this broader ecological diversity.

Looking forward, the convergence of engineering biology with an increasingly mechanistic understanding of plant–soil–microbe interactions creates a unique opportunity to design programmable rhizosphere systems. Such systems could enable responsive and sustainable agricultural practices, from real-time soil diagnostics to targeted delivery of growth-promoting functions. While significant challenges remain, the field is rapidly approaching a stage where engineered rhizobacteria can move from proof-of-concept studies to practical applications. Harnessing this potential will require not only technical innovation but also advances in regulatory frameworks and public acceptance. Together, these developments point towards a future in which the rhizosphere itself becomes an engineerable platform for improving plant productivity and resilience in a changing environment.

## Abbreviations

AHL: acyl-homoserine lactone
EngComs: engineered microbial communities
HSL: homoserine lactone
RBS: ribosome binding site
SynComs: synthetic communities
TF: transcription factor
TNT: trinitrotoluene
